# Photon-Counting CT: A Quantum Leap in Diagnostic Imaging?!

**DOI:** 10.1007/s00062-022-01145-2

**Published:** 2022-03-03

**Authors:** Martin Wiesmann

**Affiliations:** Klinik für Diagnostische und Interventionelle Neuroradiologie, RWTH Aachen, Aachen, Germany

When I started my education in neuroradiology almost 30 years ago, there was conventional x‑ray, angiography, computed tomography (CT), and magnetic resonance imaging (MRI). Truth be told, I did not expect to see a new imaging modality come into routine use before I would retire. I may have been wrong.

After 50 years of clinical use, CT is about to change in a way that feels to me like a completely new imaging modality has emerged. The first generation of CT scanners equipped with photon-counting detectors is currently being installed in hospitals for (high-end) routine use. This technology results in lower radiation dose, increased spatial resolution, and less artifacts. After many years without real progress in the field of CT imaging, this improvement is very much appreciated. But behold, I think there is much more to it.

Imagine you are “looking” at a flower garden but can detect intensity of light only. What you get, of course, is a black and white photography. Does that black and white image depict the reality accurately? In some way, yes. But everyone will readily admit that an important dimension has been missed completely: the spectral information of light, namely the various colors of the flowers! What has this got to do with photon-counting CT?

Throughout most of my professional life, I believed that CT scanners “detect” X‑ray radiation; however, technically speaking, detectors in conventional CT scanners were never able to directly react to X‑rays. As a workaround X‑rays are received by a glass layer (i.e., scintillation crystal) which converts the X‑rays to flashes of visible light. Behind the glass layer is a photodiode (comparable to what is inside digital cameras) which detects the light flashes and sums them up over a given period of time, e.g. within a few milliseconds. Thereby, the information of thousands of X‑rays is integrated into one single result and can thus be discerned from electronic noise. Is this a true depiction of reality? In some way, yes. The numerical result will be higher when the X‑rays have passed easily through water as compared to when they had to pass through bone. Remember black and white photography, anyone?

However, imaging aspects do not only depend on a material’s thickness and density but also on their type: The specific energy of X‑rays is changed depending on the material they pass through. One can utilize this in today’s conventional CT scanners, for example, by using X‑rays of different energy or multi-layered detectors (e.g. “dual energy” or current “spectral” CT). However, the fundamental principle of indirect X‑ray detection, which is based on the conversion of X‑rays into light flashes and integration of many signals into one reading, remains the same.

Wouldn’t it be wonderful, if we had a CT detector which could receive single X‑rays (photons), read their specific energy, and transmit this information so fast that it was ready again before the next X‑ray arrives? Following this true “spectral imaging”, a whole new world of information about anatomic structures and pathology might be our reward. Sounds too good to be true? Enter photon-counting CT [1]!

The technical difficulties involved here are gargantuan, and the engineers who have overcome them cannot be applauded enough! We are talking about incredibly sensitive detectors and signal transmission within the CT scanner in the low nanosecond range.

I am positively thrilled for what’s in store for us! But does that mean, photon-counting technology will have solved all our diagnostic problems in the months to come? I am afraid not. Diagnostic applications of photon-counting CT spectral imaging are still in their infancy, at best. So be prepared not to be disappointed too early. I recommend to have a look at the first cranial CT image ever published [2]. Back in 1971, it was probably very difficult to imagine what was there to come for this technology. So who knows, maybe in 10 or 20 years from now the evolution of spectral photon-counting CT will have made today’s CT images look just as outdated … Photon-counting CT is a quantum leap in the making!
